# Soil thickness reshapes the spatial patterns of soil total nitrogen concentration and stocks in a typical karst catchment

**DOI:** 10.3389/fpls.2026.1998131

**Published:** 2026-10-14

**Authors:** Yanchao Lyu, Yunchao Zhou

**Affiliations:** Institute for Forest Resources and Environment of Guizhou, Guizhou Key Laboratory of Forest Cultivation in Plateau Mountain, Guizhou Provincial Field Scientific Observation and Research Station, College of Forestry, Guizhou University, Guiyang, China

**Keywords:** decoupling effect, karst catchment, soil thickness, soil total nitrogen concentration, soil total nitrogen stocks

## Abstract

Karst regions are both ecologically fragile areas and important agricultural production zones in China, where soil nitrogen pools play a critical role in sustaining ecosystem productivity and regulating carbon–nitrogen cycling. However, highly fragmented soil cover and generally shallow soil layers hinder accurate characterization of regional soil nitrogen pools and their underlying controls. Using 2, 755 soil profiles from the Houzhaihe karst catchment in Guizhou Province, China, we integrated spatial interpolation, ANOVA, regression analysis, and structural equation modeling to examine the spatial patterns and potential drivers of soil total nitrogen (STN) concentration and soil total nitrogen (STN) stocks. The results revealed a pronounced concentration–stock divergence across both landform units and land-use types. STN concentration was approximately twice as high in karst peak-cluster areas as in depressions (3.19 vs. 1.60 g kg^-^¹), whereas STN stocks were 21.15% lower in peak-cluster areas (9.08 vs. 11.00 t ha^-^¹). Similarly, forestland exhibited the highest STN concentration (3.53 g kg^-^¹), whereas STN stocks were highest in paddy fields and upland cropland (13.40 and 10.60 t ha^-^¹), indicating greater current nitrogen storage in agricultural soils. Regression analysis further showed that STN concentration and STN stocks were not linearly correlated (*p* = 0.434) but exhibited a significant hump-shaped relationship ( $R_{adj}^{2}=0.03$, *p* < 0.001), indicating a nonlinear concentration–stock decoupling. Structural equation modeling identified soil thickness as a key structural factor potentially driving this decoupling, exerting opposite effects on STN concentration (*β* = −0.20) and STN stocks (*β* = 0.60) by increasing the effective soil mass while diluting soil nitrogen concentration. These findings support a dual concentration–stock framework for evaluating karst soil nitrogen pools and inform agricultural nitrogen management and ecological restoration.

## 1 Introduction

As global climate change and food security remain two major challenges facing human society (Hungate et al., 2003; Spiertz, 2010), nitrogen has attracted considerable attention because of its critical roles in sustaining agricultural productivity and regulating greenhouse gas emissions (Stokstad, 2016; Xiao et al., 2026). Given that soils constitute the largest nitrogen reservoir in terrestrial ecosystems (Post et al., 1985; Stevens, 2019), their nitrogen concentration and stocks directly influence soil fertility, vegetation growth, microbial processes, and ecosystem carbon–nitrogen cycling (Fowler et al., 2013; Peñuelas and Sardans, 2022; Yan et al., 2025). Karst regions are characterized by fragmented terrain, extensive bedrock exposure, shallow soils, and discontinuous soil cover (Li et al., 2021; Feng et al., 2026). Within this distinctive soil–landform system, soil total nitrogen (STN) concentration may not accurately reflect the actual magnitude of the soil nitrogen pool, as quantified by soil total nitrogen (STN) stocks. Unlike concentration, STN stocks integrate nitrogen concentration with the amount of soil present per unit land area. This distinction is particularly important in karst landscapes, where strong spatial heterogeneity in soil thickness, bedrock exposure, and coarse-fragment content can produce large differences in effective soil mass and, consequently, in the size of the soil nitrogen pool even at similar STN concentrations. Simultaneous assessment of STN concentration and STN stocks is therefore essential for accurately characterizing the spatial patterns and ecological functions of soil nitrogen in karst regions.

Although STN stocks serve as a key metric of the size of the soil nitrogen pool, they have received comparatively limited attention. On the one hand, soil nitrogen pools have long been regarded as slowly cycling reservoirs dominated by stable and recalcitrant forms, leading their roles in nutrient supply and environmental processes to be underestimated (Galloway et al., 2013; Bingham and Cotrufo, 2016; Morseletto, 2019). However, increasing evidence indicates that soil nitrogen pools contain substantial amounts of structurally simple, actively cycling nitrogen fractions that are sensitive to environmental change (Chenu et al., 2019; Smith et al., 2020; Petersson et al., 2024). Soil nitrogen pools may thus function both as long-term sources of plant-available nitrogen and as regulators of greenhouse-gas emissions through processes such as nitrification and denitrification (Boyle, 2017; Bowles et al., 2018; Ding et al., 2026). On the other hand, STN concentration reflects nitrogen enrichment per unit soil mass, whereas STN stocks quantify the cumulative amount of nitrogen per unit land area within a defined soil depth (Mason et al., 2022; Wang et al., 2025). Although these two metrics are related, they describe different dimensions of soil nitrogen status. In non-karst regions with well-developed and relatively continuous soils, STN concentration and STN stocks may exhibit close correspondence (Li et al., 2020; Almaraz et al., 2025), which partly explains why soil nitrogen assessments have more commonly relied on concentration rather than stocks. In karst regions, however, soil thickness and soil-cover continuity are highly heterogeneous (Buckerfield et al., 2019a; Zhang et al., 2021). Assessments based solely on STN concentration may therefore either underestimate or overestimate the actual size of the soil nitrogen pool.

Despite growing interest in soil nitrogen cycling, understanding of soil nitrogen pools in karst regions remains incomplete. This is particularly true at the karst catchment scale, where landforms and land-use types are highly interspersed. Whether STN concentration and STN stocks exhibit consistent spatial differentiation, and how these two metrics correspond to one another, have not yet been systematically evaluated using large, spatially detailed datasets. Clarifying these relationships would help avoid biased assessments of soil nitrogen pool size based solely on STN concentration, facilitate the identification of soil units with high STN stocks, and provide a scientific basis for agricultural nitrogen management, nitrogen-loss risk control, and catchment-scale carbon–nitrogen cycling research (Conley et al., 2009; Vitousek et al., 2009; Zhang et al., 2024; Chen et al., 2026). An integrated analytical framework incorporating both landform and land use is therefore needed to quantify the relationships between environmental factors and STN concentration and STN stocks and to identify the structural controls underlying the spatial differentiation of soil nitrogen pools in karst regions.

Accordingly, this study investigated the Houzhaihe karst catchment using a large-scale soil-profile survey and integrated spatial interpolation, analysis of variance, regression analysis, and structural equation modeling to examine the spatial differentiation and environmental controls of STN concentration and STN stocks. The specific objectives were to: (i) characterize the spatial patterns of STN concentration and STN stocks across peak-cluster–depression landform units and different land-use types and identify soil units with high STN stocks; (ii) evaluate the relationship between STN concentration and STN stocks and determine whether they exhibit concentration–stock decoupling, defined here as a mismatch between their spatial or categorical patterns; and (iii) quantify the direct and indirect effects of soil organic carbon (SOC), bedrock exposure ratio, soil thickness, soil gravel content, and other environmental factors on STN concentration and STN stocks, thereby identifying potential key mechanisms underlying the spatial differentiation of soil nitrogen pools in karst landscapes. The findings are expected to advance the theoretical understanding of the spatial differentiation and environmental controls of soil nitrogen within karst catchments and provide scientific support for soil nutrient assessment, agricultural nitrogen management, and ecological restoration in karst regions.

## 2 Materials and methods

### 2.1 Study area

The study area, the Houzhaihe catchment, is located in Puding County, Guizhou Province, southwestern China (**Figure 1A**), and represents a typical plateau karst catchment. The catchment lies near the watershed divide between the Yangtze and Pearl River basins and extends from 105°40′43″ to 105°48′02″ E and from 26°12′29″ to 26°17′15″ N, covering an area of approximately 75 km². Elevation ranges from 1, 189 to 1, 519 m, with a relative relief of approximately 250–300 m. The terrain is generally higher in the southeast and lower in the northwest (**Figure 1B**). The region has a humid subtropical monsoon climate, with a mean annual temperature of approximately 15.1 °C, mean annual precipitation of approximately 1, 378 mm, and a frost-free period of approximately 300 days. Annual precipitation is strongly seasonal, with most rainfall occurring during the wet season (Qin et al., 2019; Bai and Zhou, 2020).

**Figure 1:**
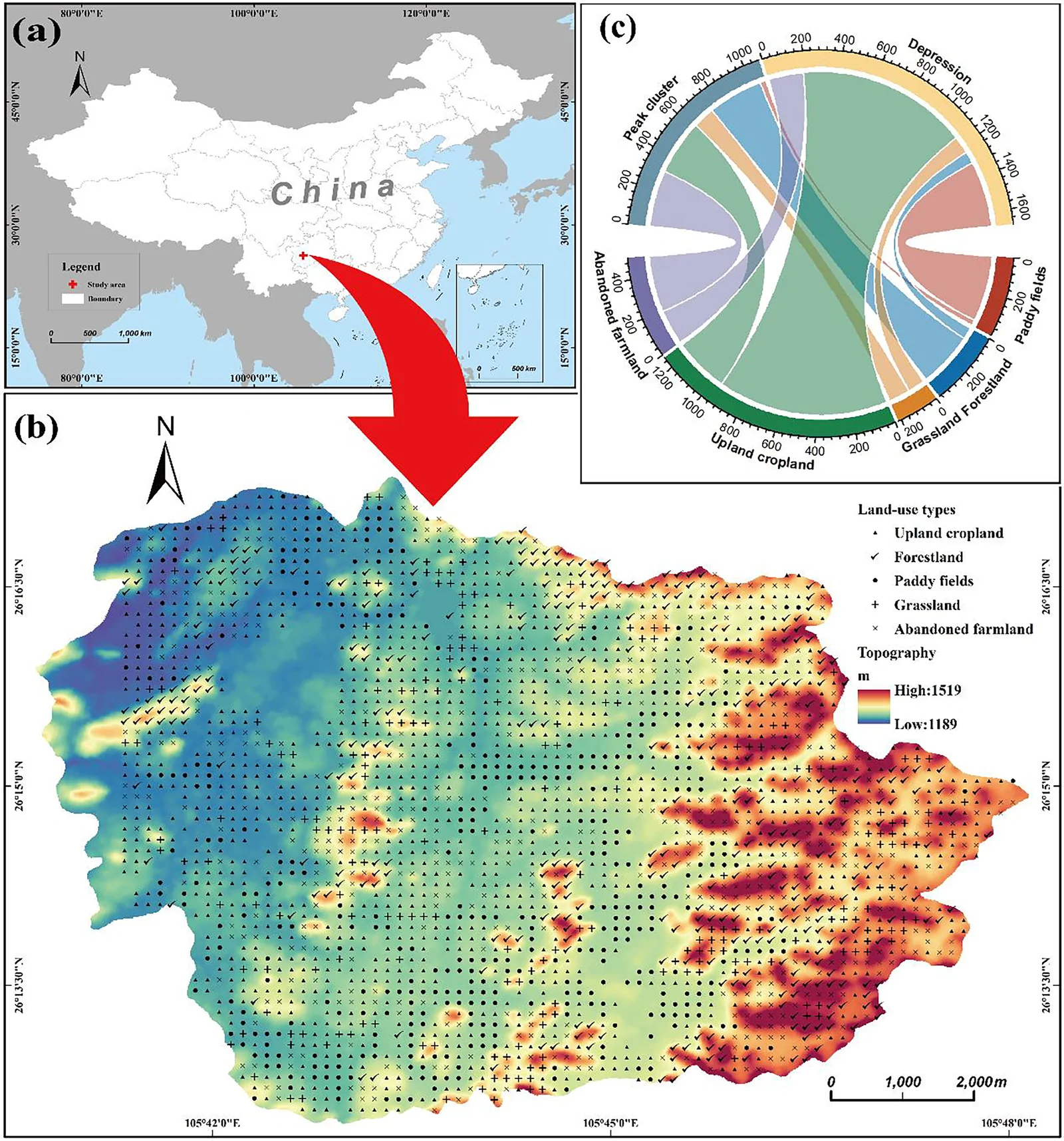
Overview of the Houzhaihe catchment. **(A)** Location of the Houzhaihe catchment in Guizhou Province, Southwest China; **(B)** Soil sampling design overlaid on a digital elevation model (DEM), showing the spatial distribution of sampling grids and soil profile sites; **(C)** Correspondence between karst landform units and land-use types. The values shown in the chord diagram represent the number of sampling grids, each with a size of 150 × 150 m.

Carbonate rocks are extensively exposed throughout the Houzhaihe catchment. More than 90% of the catchment is underlain by carbonate rocks of the Middle Triassic Guanling Formation, whose principal lithologies are limestone, dolomite, and argillaceous limestone. Intense karstification has shaped the catchment into a well-developed peak-cluster–depression landscape, in which peak clusters are interspersed with karst depressions. Land use is diverse and primarily includes forestland, grassland, abandoned farmland, upland cropland, and paddy fields. Forestland, grassland, and abandoned farmland are mainly distributed across peak clusters and hillslopes, whereas upland cropland and paddy fields are concentrated in depressions and on gentle slopes (**Figure 1C**). The spatial interspersion of land-use types and landforms provides the physical basis for the high spatial heterogeneity of material and energy distributions within the catchment.

### 2.2 Experimental design and soil sampling

To systematically investigate the spatial differentiation of STN concentration and STN stocks across the Houzhaihe catchment, a field-based soil-profile survey was conducted in autumn 2015 using a regular-grid sampling design. A regular sampling grid with a resolution of 150 m × 150 m was generated for the study area using ArcGIS, and the center of each grid cell was designated as the preferred sampling location. Sampling points were located in the field using a global positioning system (GPS). Where permanent buildings, water bodies, or other obstacles prevented sampling at the designated location, an alternative site with similar landform and land-use characteristics was selected nearby. In total, 2, 755 soil profiles were established and surveyed, covering the principal landform units and land-use types within the catchment.

At each sampling point, a soil profile was excavated and the actual soil thickness was recorded. Where the soil thickness was ≥100 cm, the profile was excavated to a depth of 100 cm. Where the soil thickness was <100 cm, excavation continued to the underlying bedrock or parent material, and the actual soil thickness was used as the sampling depth. Soil samples were collected by depth interval. Within the upper 0–20 cm, samples were collected at 5-cm intervals (0–5, 5–10, 10–15, and 15–20 cm), whereas samples from 20 to 100 cm were collected at 10-cm intervals (20–30, 30–40, and 90–100 cm). Approximately 1 kg of soil was collected from each depth interval. Corresponding undisturbed samples for bulk-density determination were collected using stainless-steel cores. All samples were labeled and transported to the laboratory for subsequent analysis.

The geographic coordinates, elevation, slope, land-use type, soil thickness, bedrock exposure ratio (BR), and fractional vegetation cover of each sampling point were recorded during field sampling. Soil thickness was determined by combining direct profile measurements with steel-rod probing. The BR assigned to each soil profile represented the areal proportion of exposed bedrock within the corresponding 150 × 150 m sampling grid. Both BR and fractional vegetation cover were derived by integrating field observations, remote-sensing image interpretation, and spatial analysis in ArcGIS. Based on field observations and remote-sensing imagery, land use was classified into forestland, grassland, abandoned farmland, upland cropland, and paddy fields. Landform units were classified as peak clusters or depressions according to their topographic characteristics.

### 2.3 Laboratory analyses

After transportation to the laboratory, plant roots, litter, and large gravel fragments were removed from the soil samples. The samples were then air-dried, ground, and sieved for physicochemical analyses. STN concentration was determined using laser-induced breakdown spectroscopy. Briefly, air-dried soil samples were compressed into circular pellets, after which a high-energy pulsed laser was focused on the sample surface to generate plasma. The target elements in the sample were excited and emitted characteristic spectral lines, from which STN concentration was estimated based on the spectral signals. The results were expressed in g kg^-^¹ (Xu et al., 2020). After the nitrogen concentration of each sampled soil layer had been determined, the arithmetic mean across all layers within a profile was calculated and used as the profile-scale STN concentration, representing the overall nitrogen concentration at that sampling point. This profile-scale metric was used in subsequent analyses to provide an integrated measure of nitrogen enrichment for each soil profile and to enable comparison with profile-scale STN stocks at the same sampling locations.

SOC concentration was determined using the potassium dichromate oxidation method with external heating (Even et al., 2025). Samples collected for bulk-density determination were oven-dried at 105 °C to a constant mass. Soil bulk density was then calculated as the oven-dry soil mass per unit core volume and expressed in g cm^-^³. Soil gravel content was calculated as the mass percentage of particles >2 mm relative to the total sample mass.

STN stocks were calculated from STN concentration, soil bulk density, soil-layer thickness, bedrock exposure ratio, and soil gravel content. The nitrogen stock of an individual soil layer was calculated using Equation 1:

(1) $$STN\_stocks_{i}=STN_{i}\times BD_{i}\times T_{i}\times (1-BR)\times (1-G_{i})\times 0.1$$

where $STN\_stocks_{i}$ is the nitrogen stock in soil layer i (t ha^-^¹); $STN_{i}$ is the soil total nitrogen concentration in soil layer i (g kg^-^¹); $BD_{i}$ is the soil bulk density in soil layer i (g cm^-^³); $T_{i}$ is the thickness of soil layer i (cm); BR is the bedrock exposure ratio within the 150 × 150 m sampling grid corresponding to the soil profile; $G_{\dot{l}}$ is the gravimetric proportion of gravel in soil layer i; and 0.1 is the unit-conversion factor. Profile-scale STN stocks were obtained by summing nitrogen stocks across all sampled soil layers, as shown in Equation 2:

(2) $$STN\_stocks=\sum_{i=1}^{n}STN\_stocks_{i}$$

where STN_stocks is the profile-scale soil total nitrogen stock (t ha^-^¹), and n is the actual number of sampled soil layers. For profiles with a soil thickness of <100 cm, STN stocks were calculated only over the actual soil depth.

### 2.4 Statistical analyses

Statistical analyses focused on the spatial differentiation and environmental controls of STN concentration and STN stocks. All statistical analyses and model fitting were conducted in R version 4.3.2, whereas spatial analyses and map production were performed using ArcGIS 10.8. Ordinary kriging was first applied to generate continuous spatial distributions of STN concentration and STN stocks across the catchment. The interpolated distributions were subsequently compared with the elevation map to identify spatial differences in soil nitrogen patterns among landform units. One-way analysis of variance was used to test differences in STN concentration and STN stocks among landform units and land-use types. When the overall difference was significant at p < 0.05, Tukey’s honestly significant difference test was performed for multiple pairwise comparisons to determine differences among individual groups.

Linear and quadratic regression models were fitted to examine the relationship between STN concentration and STN stocks and to identify potential nonlinear associations and concentration–stock decoupling. Model explanatory power and goodness of fit were evaluated using the adjusted coefficient of determination, overall model significance, and the significance of individual regression coefficients.

To further elucidate the mechanisms underlying the spatial differentiation of soil nitrogen, structural equation modeling was performed using the ‘lavaan’ package in R (Rosseel, 2012). Based on the characteristics of soil development in karst landscapes and the *a priori* conceptual framework, environmental variables including SOC concentration, soil thickness, soil bulk density, bedrock exposure ratio, fractional vegetation cover, and soil gravel content were incorporated into the model to identify their direct and indirect effects on STN concentration and STN stocks. Standardized path coefficients were used to quantify the strength and direction of the relationships among variables. Overall model fit was evaluated using the chi-square test (*χ^2^*), comparative fit index (CFI), and root mean square error of approximation (RMSEA). Because soil thickness, soil bulk density, soil gravel content, and bedrock exposure ratio are direct components of the STN-stock calculation, their modeled associations with STN stocks partly reflect mathematical coupling. Accordingly, SEM path coefficients were interpreted as conditional multivariate associations rather than as independent evidence of ecological causality.

## 3 Results

### 3.1 Spatial patterns of soil nitrogen in the Houzhaihe catchment

Soil nitrogen exhibited pronounced spatial variation across the Houzhaihe catchment (**Figures 2A, C**). The distribution of high and low STN concentration values also corresponded closely to the peak-cluster–depression topographic pattern (**Figures 1B**, **2A**). Ordinary kriging showed that STN concentration was distributed in spatially heterogeneous patches across the study area. Areas with high STN concentration were mainly associated with high-relief peak clusters along the catchment margins and with isolated peak-like uplands, whereas low values occurred predominantly in the relatively low-lying depressions within the interior of the catchment. Comparisons between landform units further showed that STN concentration was significantly higher in peak clusters than in depressions (**Figure 2B**), averaging 3.19 and 1.60 g kg^-^¹, respectively. STN concentration in peak clusters was approximately twice that in depressions, indicating greater nitrogen enrichment per unit soil mass in peak-cluster soils.

**Figure 2:**
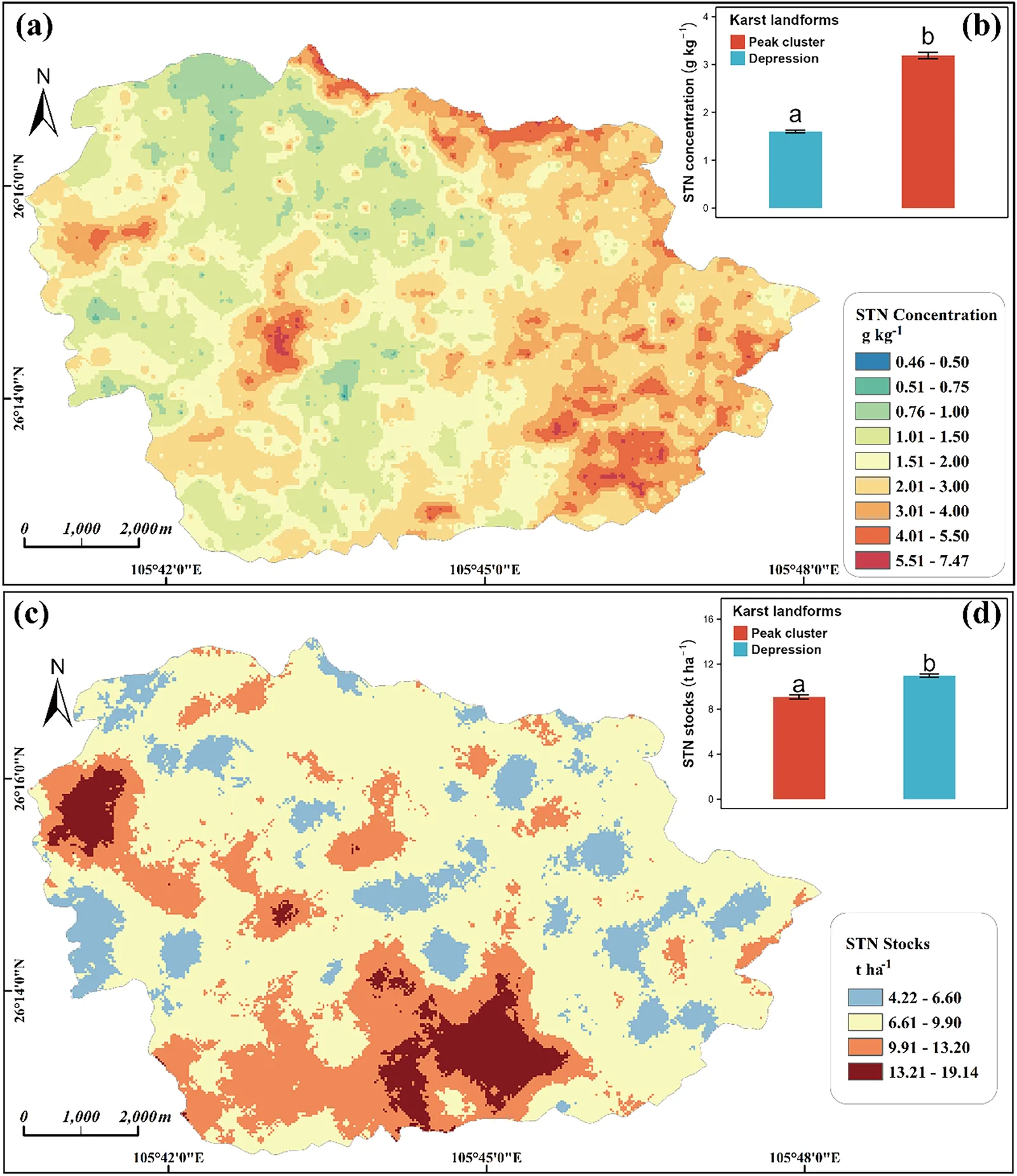
Spatial variation of soil nitrogen in the Houzhaihe catchment. Kriging-interpolated maps and landform-level comparisons jointly reveal contrasting spatial patterns between STN concentration and STN stocks. **(A)** Spatial distribution of STN concentration based on kriging interpolation; **(B)** differences in STN concentration between karst landform units; **(C)** spatial distribution of STN stocks based on kriging interpolation; and **(D)** differences in STN stocks between karst landform units. Data in panels **(B, D)** are *means* ± *SE*. Different lowercase letters indicate significant differences between peak clusters and depressions based on Tukey’s HSD test at *p* < 0.05. Error bars represent standard errors (*SE*).

In contrast to STN concentration, STN stocks exhibited an opposing pattern of differentiation between landform units. Spatial interpolation showed that areas with high STN stocks were more frequently distributed in the low-lying depressions within the catchment interior (**Figure 2C**). STN stocks were significantly higher in depressions than in peak clusters (**Figure 2D**), averaging 11.00 and 9.08 t ha^-^¹, respectively. STN stocks in depressions were 21.15% higher than those in peak clusters, indicating that depression soils contained more nitrogen per unit land area despite their lower STN concentration. Overall, STN concentration and STN stocks exhibited contrasting spatial patterns, indicating that assessments of soil nitrogen status based solely on STN concentration may underestimate the nitrogen-accumulation capacity of depression soils.

### 3.2 Soil nitrogen patterns among land-use types

Soil nitrogen differed significantly among the five major land-use types in the Houzhaihe catchment (**Figure 3**). Forestland had the highest STN concentration, averaging 3.53 g kg^-^¹, followed by abandoned farmland and grassland, with mean values of 3.06 and 2.91 g kg^-^¹, respectively (**Figure 3A**). Upland cropland and paddy fields had the lowest STN concentration, averaging 1.60 and 1.41 g kg^-^¹, respectively. Tukey’s HSD test showed that STN concentration in forestland, abandoned farmland, and grassland was significantly higher than that in upland cropland and paddy fields (*p* < 0.05). Specifically, STN concentration in forestland was 150.35% higher than that in paddy fields and 120.63% higher than that in upland cropland. STN concentration in abandoned farmland and grassland was also 117.02% and 106.38% higher, respectively, than that in paddy fields. Overall, forestland, abandoned farmland, and grassland formed a group characterized by relatively high STN concentration, whereas upland cropland and paddy fields formed a group with relatively low STN concentration.

**Figure 3:**
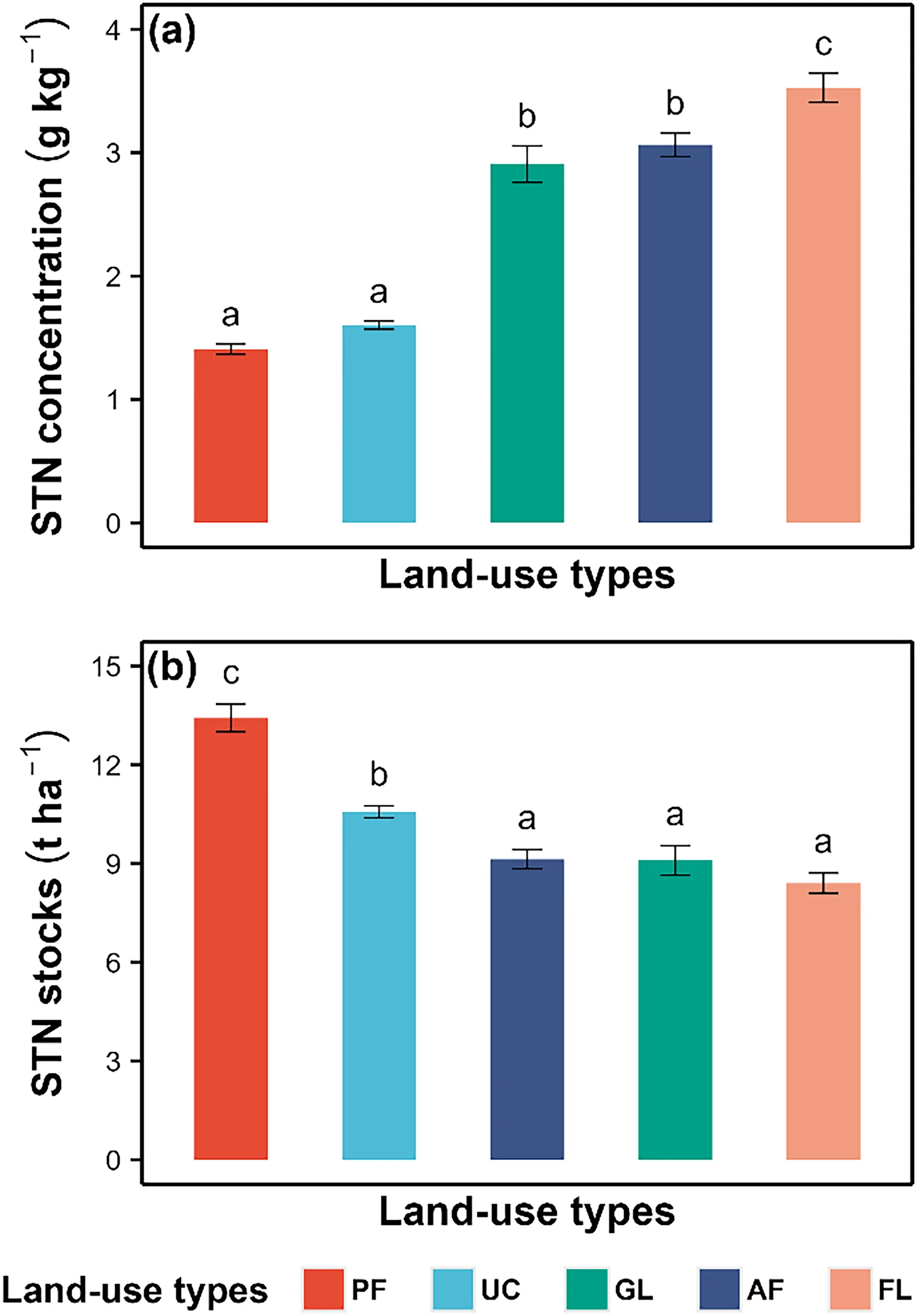
Differentiation patterns of soil nitrogen across land-use types in the Houzhaihe catchment. **(A)** STN concentration and **(B)** STN stocks among different land-use types. The abbreviations PF, UC, GL, AF and FL represent paddy fields, upland cropland, grassland, abandoned farmland and forestland, respectively. Data in panels **(A, B)** are *means* ± *SE*. Different lowercase letters denote significant differences among land-use types according to Tukey’s HSD test at *p* < 0.05. Error bars represent standard errors (*SE*).

STN stocks, however, exhibited a pattern that was nearly opposite to that of STN concentration (**Figure 3B**). Paddy fields had the highest STN stocks, averaging 13.40 t ha^-^¹, followed by upland cropland at 10.60 t ha^-^¹. By comparison, abandoned farmland, grassland, and forestland had relatively low STN stocks, averaging 9.14, 9.10, and 8.41 t ha^-^¹, respectively. Tukey’s HSD test showed that STN stocks in paddy fields and upland cropland were significantly higher than those in abandoned farmland, grassland, and forestland (*p* < 0.05). STN stocks in paddy fields were 59.33% higher than those in forestland and 47.25% higher than those in grassland, whereas STN stocks in upland cropland were 26.04% and 16.48% higher than those in forestland and grassland, respectively. Thus, paddy fields and upland cropland formed a group with relatively high STN stocks, whereas forestland, grassland, and abandoned farmland formed a group with relatively low STN stocks. Notably, STN concentration and STN stocks did not vary consistently among land-use types. Although forestland and grassland had relatively high STN concentration, their STN stocks did not increase correspondingly. In contrast, paddy fields and upland cropland exhibited higher STN stocks despite their relatively low STN concentration.

### 3.3 Relationship between STN concentration and STN stocks in karst soils

Because STN concentration and STN stocks did not exhibit consistent patterns among either landform units or land-use types (**Figures 2**, **3**), linear and quadratic regression analyses were performed to further examine their relationship within the Houzhaihe karst catchment. No significant linear relationship was detected between STN concentration and STN stocks (**Figure 4A**; *p* = 0.434). In contrast, quadratic regression revealed a significant nonlinear relationship between the two variables (**Figure 4B**). The fitted equation was *y* = 8.01+1.89*x*-0.255*x^2^*, where *x* represents STN concentration and *y* represents STN stocks. Both the overall model and the quadratic term were highly significant ( $p_{model}$ < 0.001 and $p_{\text{quadratic}}$ < 0.001), indicating a significant quadratic relationship between STN concentration and STN stocks.

**Figure 4:**
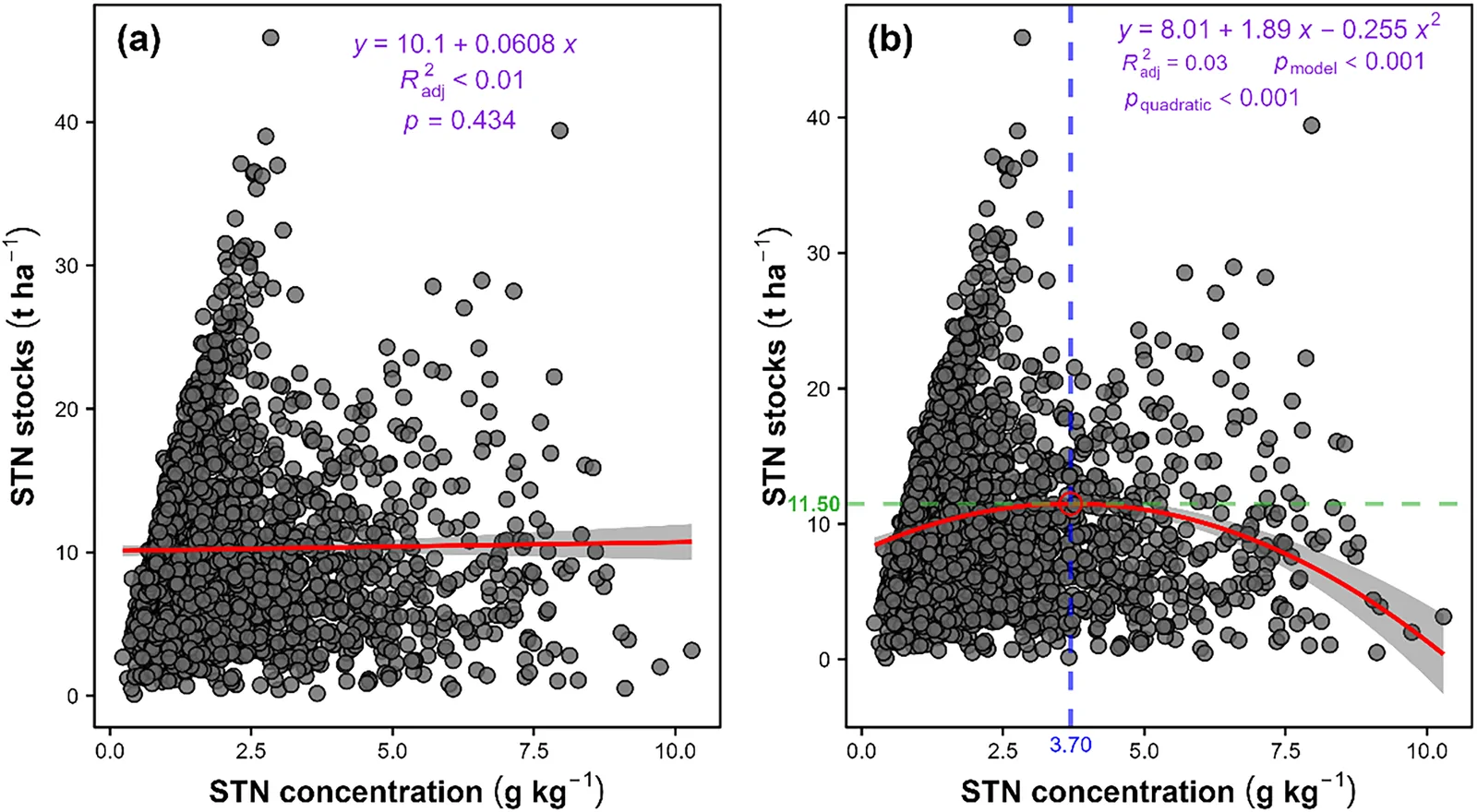
The relationship between STN concentration and STN stocks in the Houzhaihe karst catchment. **(A)** Linear regression model. **(B)** Quadratic regression model. The red straight line in panel **(A)** and the red curve in panel **(B)** represent the linear and quadratic regression fits, respectively. In both panels, shaded bands indicate 95% confidence intervals; regression equations, adjusted *R²*, and *p* values are shown in each panel. For the quadratic model, the blue vertical dashed line and green horizontal dashed line indicate the STN concentration and STN stocks at the vertex of the regression curve, respectively. The red circle denotes the vertex at which the predicted STN stocks reach the maximum value, corresponding to a STN concentration of 3.70 g kg^-^¹ and STN stocks of 11.50 t ha^-^¹.

According to the fitted curve, STN stocks initially increased and subsequently decreased as STN concentration increased (**Figure 4B**). The fitted quadratic relationship reached an empirical turning point at an STN concentration of 3.70 g kg^-^¹, corresponding to a maximum predicted STN stock of 11.50 t ha^-^¹. Below 3.70 g kg^-^¹, increasing STN concentration corresponded to increasing STN stocks. Once STN concentration exceeded 3.70 g kg^-^¹, however, STN stocks did not continue to increase but instead showed a declining trend. These results demonstrate that high STN concentration does not necessarily correspond to high STN stocks in the Houzhaihe karst catchment, indicating a clear concentration–stock decoupling pattern.

Although the quadratic model was highly significant, its adjusted coefficient of determination was low ( $R_{adj}^{2}$ = 0.03), indicating that STN concentration explained only a small proportion of the variation in STN stocks. Accordingly, the empirical turning point of 3.70 g kg^-^¹ should not be interpreted as a general ecological threshold. The scatterplot also showed substantial variation in STN stocks among soils with identical or similar STN concentration values. This weak curvilinear relationship indicates that STN stocks cannot be reliably inferred from STN concentration alone in karst ecosystems.

### 3.4 Potential environmental controls on soil nitrogen in the Houzhaihe karst catchment

The structural equation model showed that STN concentration and STN stocks were associated with multiple environmental factors, although their dominant structural pathways differed markedly (**Figure 5A**). The model exhibited a good fit to the data (*χ^2^* = 1.36, *p* = 0.51, CFI = 1.00, and RMSEA = 0) and explained 76.3% of the variation in STN concentration and 60.3% of the variation in STN stocks.

**Figure 5:**
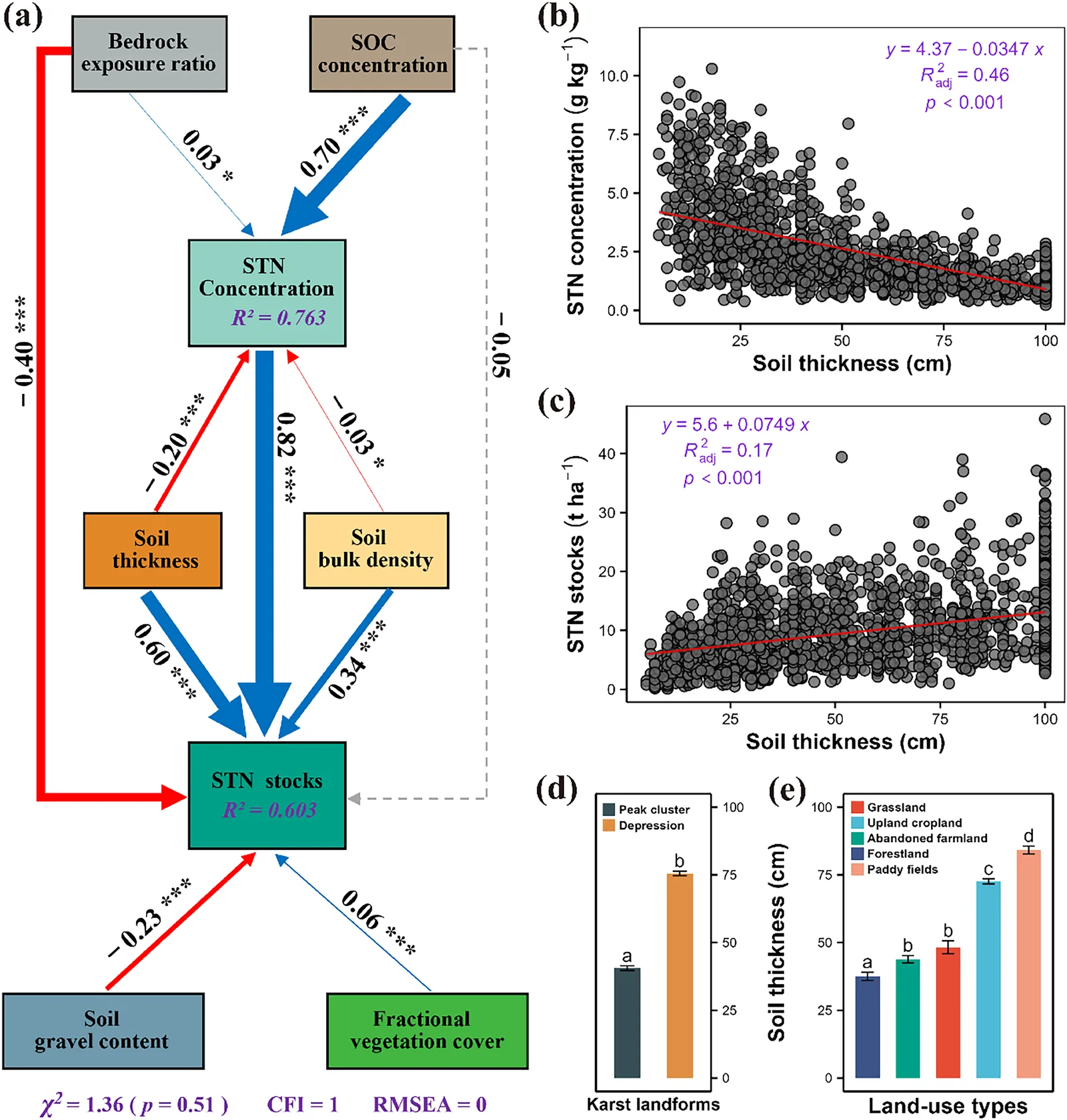
Environmental controls on soil total nitrogen in the Houzhaihe karst catchment. **(A)** Structural equation model. Values beside arrows are standardized path coefficients, with *, **, and *** indicating *p* < 0.05, *p* < 0.01, and *p* < 0.001, respectively. Arrow width indicates the strength of the effect; blue and red arrows represent positive and negative effects, respectively. $R^{2}$ values indicate the proportion of variance explained for endogenous variables. Model fit indices are shown at the bottom of panel **(A)**. Linear relationships of STN concentration **(B)** and STN stocks **(C)** with soil thickness. Red lines represent fitted linear regressions, and light-gray shaded bands represent 95% confidence intervals. Differences in soil thickness among karst landform types **(D)** and land-use types **(E)**. Bars represent *means* ± *SE*; different lowercase letters indicate significant differences (Tukey’s HSD, *p* < 0.05).

STN concentration was primarily associated with SOC concentration and soil thickness (**Figure 5A**). Within the SEM, SOC concentration exhibited the strongest positive effect on STN concentration (*β* = 0.70), whereas soil thickness had a significant negative effect (*β* = −0.20). Compared with STN concentration, STN stocks were influenced by a broader range of environmental factors. STN concentration exerted the strongest positive direct effect on STN stocks (*β* = 0.82), indicating that nitrogen concentration remained an important determinant of the amount of nitrogen stored per unit area. In addition to STN concentration, soil thickness had a significant positive effect on STN stocks (*β* = 0.60), with an effect size second only to that of STN concentration. Soil bulk density also had a significant positive effect on STN stocks (*β* = 0.34). By contrast, bedrock exposure ratio and soil gravel content had significant negative effects on STN stocks, with standardized path coefficients of −0.40 and −0.23, respectively.

Notably, soil thickness was the only factor that exerted relatively strong effects in opposite directions on STN concentration and STN stocks. In comparison, the effects of bedrock exposure ratio and soil bulk density on STN concentration were negligible, with path coefficients of only 0.03 and −0.03, respectively. Linear regression analyses further revealed opposing relationships between soil thickness and the two soil nitrogen metrics: STN concentration decreased significantly with increasing soil thickness (**Figure 5B**), whereas STN stocks increased significantly with increasing soil thickness (**Figure 5C**).

Soil thickness also differed significantly among landform units and land-use types (**Figures 5D, E**). Soils in depressions were significantly thicker than those in peak clusters, averaging 75.5 and 40.5 cm, respectively. Paddy fields and upland cropland also had thicker soils than grassland, abandoned farmland, and forestland, with mean thicknesses of 84.2 and 72.6 cm compared with 48.2, 43.8, and 37.5 cm, respectively. These results further identify soil thickness as a key structural factor linking the divergent behavior of STN concentration and STN stocks, because it simultaneously affects concentration patterns and the spatial accumulation of soil nitrogen.

## 4 Discussion

### 4.1 Farmland soils exhibit a relatively high nitrogen-storage capacity

In contrast to the pattern observed for STN concentration, STN stocks were significantly higher in paddy fields and upland cropland than in forestland, grassland, and abandoned farmland. This finding indicates that STN concentration and STN stocks represent different dimensions of soil nitrogen status. A high STN concentration does not necessarily correspond to a large STN stock, whereas farmland soils with relatively low STN concentration may still store substantial amounts of nitrogen per unit land area. Here, the term “nitrogen-storage capacity” is used in a broad sense, encompassing both current area-based nitrogen storage and the potential for further accumulation, rather than referring to a measured maximum storage limit. Therefore, STN stocks in karst regions cannot be accurately assessed on the basis of STN concentration alone.

This discrepancy is closely associated with the geomorphic distribution of land-use types within the study area (Buckerfield et al., 2019b; Wang et al., 2020). Forestland, grassland, and abandoned farmland are distributed mainly across peak clusters and hillslopes, whereas paddy fields and upland cropland occur predominantly in low-lying depressions and on gentle slopes (**Figure 1C**). Forestland, grassland, and abandoned farmland generally have greater vegetation cover and litter inputs and experience less anthropogenic disturbance, favoring the accumulation of organic matter and nitrogen in surface and shallow soils (Jackson et al., 2017; Chien and Krumins, 2023; Yang et al., 2026). These conditions may account for their relatively high STN concentrations. However, peak-cluster areas are generally characterized by shallow soils, high bedrock exposure, and high gravel contents, which restrict the effective soil mass available for nitrogen storage. Consequently, the high STN concentration observed in forestland, grassland, and abandoned farmland did not translate into correspondingly high STN stocks.

By comparison, paddy fields and upland cropland had significantly higher STN stocks despite their relatively low STN concentrations. This pattern may partly reflect agricultural management practices. Fertilization, irrigation, and organic material inputs increase external nitrogen inputs and influence the accumulation and transformation of soil nitrogen (Gautam et al., 2025; Reis Ely et al., 2025). The pattern is also related to the predominant occurrence of farmland in karst depressions. Depressions generally have thicker and more spatially continuous soils (Zeng et al., 2020), providing a larger soil volume in which nitrogen can accumulate. Thus, the relatively high STN stocks in farmland soils do not simply reflect high nitrogen concentrations but instead represent the combined outcome of nitrogen inputs, topographic setting, and soil thickness. These findings demonstrate that both land use and its geomorphic context should be considered when investigating the spatial distribution of soil nitrogen pools within karst catchments.

Farmland soils may also have considerable potential for further increases in STN stocks in karst regions. Forestland and grassland exhibited a combination of high STN concentration and relatively low STN stocks, suggesting that their soil nitrogen pools may be approaching a comparatively stable state and that the scope for further increases in area-based nitrogen storage may be limited. In contrast, STN concentration in paddy fields and upland cropland remained relatively low, indicating potential for increasing both nitrogen concentration per unit soil mass and total nitrogen stocks. Practices such as increasing organic fertilizer inputs, cultivating green manure crops, and adopting conservation tillage may improve soil nitrogen retention and expand soil nitrogen pools in farmland (Wang et al., 2016; Dos Santos et al., 2021). Moreover, where farmland is converted to forestland or grassland under ecological restoration programs, increases in STN concentration may contribute to further gains in STN stocks (Meena et al., 2018; Hong et al., 2023). Farmland soils may therefore represent not only current hotspots of soil nitrogen storage in karst landscapes but also an important potential contributor to future increases in soil nitrogen pools resulting from land-use change and ecological restoration.

### 4.2 Soil thickness is a key factor underlying the decoupling between STN concentration and STN stocks in karst soils

In the Houzhaihe catchment, STN concentration and STN stocks exhibited markedly inconsistent patterns at both the landform-unit and land-use levels. Regression analysis further showed that their relationship was not simply linear. Within the lower range of STN concentration, STN stocks generally increased with increasing concentration. At higher concentrations, however, STN stocks did not continue to increase and instead tended to decline. These findings indicate that high STN concentration does not necessarily correspond to high STN stocks in karst regions, revealing a clear concentration–stock decoupling pattern.

Importantly, this nonlinear relationship does not imply that an increase in STN concentration itself causes STN stocks to decline. Rather, high STN concentrations frequently occur in soils characterized by shallow profiles, high bedrock exposure, and high gravel contents. The structural equation model showed that, after soil thickness, bedrock exposure ratio, soil bulk density, and soil gravel content were considered simultaneously, STN concentration remained significantly and positively associated with STN stocks. STN concentration therefore remains a fundamental component of area-based nitrogen stocks. The observed decoupling is more likely to result from interactions among multiple factors than from a loss of importance of STN concentration itself (Du and De Vries, 2025). At the same time, soil thickness was negatively associated with STN concentration but positively associated with STN stocks, suggesting that it is an important structural factor contributing to their contrasting spatial patterns.

Soil thickness was significantly and negatively associated with STN concentration. One explanation is that thick soil profiles contain a greater proportion of subsoil, where organic matter and nitrogen concentrations are generally lower than in surface soil (Szatmári et al., 2019; Wiesmeier et al., 2019). When deeper soil layers with lower nitrogen concentrations are included in the calculation for the entire profile, they reduce profile-mean STN concentration, producing a vertical dilution effect (Yoo et al., 2006; Li et al., 2024). In addition, areas with thicker soils in karst catchments are more likely to be cultivated. Long-term cultivation, crop harvesting, and tillage disturbance may accelerate soil organic matter mineralization and remove nitrogen through harvested biomass, further reducing STN concentration (Chenu et al., 2019; van der Voort et al., 2023). Consequently, farmland soils in depressions do not necessarily have high STN concentration despite their greater soil thickness.

In contrast, greater soil thickness contributes strongly to the accumulation of STN stocks. STN stocks depend on both nitrogen content per unit soil mass and the effective soil mass per unit land area. As soil thickness increases, the volume and mass of soil per unit land area also increase, providing a larger soil reservoir in which nitrogen can be retained (Gelaw et al., 2014; Anuo et al., 2025). Therefore, even where profile-mean STN concentration is relatively low, thick soils may contain large STN stocks because of their greater effective soil mass (Stockmann et al., 2013; Dignac et al., 2017). Bedrock exposure ratio, soil gravel content, and soil bulk density similarly affect STN stocks by altering the effective amount of soil present per unit land area (Liu et al., 2019; Li et al., 2023).

Nevertheless, soil thickness, soil bulk density, soil gravel content, and bedrock exposure ratio are themselves components of the calculation of STN stocks. Their statistical relationships with STN stocks therefore contain information about ecological processes as well as a degree of mathematical coupling (Ibrahim et al., 2026). In this context, the structural equation model primarily reveals conditional associations and the relative contributions of the included variables rather than providing independent evidence of ecological causality. Despite this limitation, the results identify soil thickness as a key link between nitrogen enrichment per unit soil mass and nitrogen accumulation per unit land area. Soil thickness therefore provides an important structural basis for explaining the spatial decoupling of STN concentration and STN stocks in karst regions.

### 4.3 Implications for the assessment and management of soil nitrogen pools in karst regions

The results demonstrate that evaluations of soil nitrogen in karst regions should explicitly distinguish between STN concentration and STN stocks. In conventional soil nutrient assessment systems, STN concentration is commonly used as an indicator of soil nitrogen status and fertility (Hu et al., 2024; Gao et al., 2026). It effectively reflects nitrogen enrichment per unit soil mass and the potential availability of nitrogen for plant growth. For regional nitrogen-pool estimation and catchment-scale nitrogen-cycle studies, however, STN stocks provide a more direct measure of the actual size of the soil nitrogen pool (Gruber and Galloway, 2008; Galloway et al., 2013).

This distinction is particularly important in karst regions, where soil thickness and bedrock exposure are highly heterogeneous. Assessments based solely on STN concentration may incorrectly identify peak-cluster hillslopes, forestland, and grassland as areas with large nitrogen stocks while overlooking the greater effective soil mass and area-based nitrogen accumulation in farmland soils within depressions. Soil nitrogen assessment in karst landscapes should therefore move beyond a single concentration-based indicator toward an integrated framework combining STN concentration and STN stocks (Breure et al., 2025). STN concentration remains important for evaluating soil fertility and vegetation restoration (Hu et al., 2024), whereas STN stocks should receive greater emphasis in regional nitrogen-pool accounting, carbon–nitrogen balance assessments, and catchment-scale nitrogen-cycle research (Gruber and Galloway, 2008; Galloway et al., 2013).

From a management perspective, our results directly show that karst farmland, particularly paddy fields and upland cropland, has relatively high STN stocks, indicating that these soils constitute important regional nitrogen reservoirs. Previous studies further suggest that, because the soil–bedrock–groundwater continuum in karst landscapes is highly connected, excessive nitrogen fertilization or inappropriate cultivation practices may increase the risks of nitrate leaching and gaseous nitrogen losses (Li et al., 2017; Wang et al., 2025). The objective of farmland nitrogen management should therefore not be limited to increasing soil nitrogen levels but should instead seek a balance among crop nitrogen supply, soil nitrogen retention, and environmental losses. Accordingly, management practices proposed in previous studies, such as increasing organic fertilizer and green manure inputs, adopting conservation tillage, and optimizing the timing and rate of nitrogen fertilization, may improve nitrogen-use efficiency and soil nitrogen retention while reducing the risk of agricultural nonpoint-source pollution (Xu and Zhang, 2021; Wang et al., 2022).

The broader applicability of these findings should nevertheless be interpreted with some caution. This study was conducted within a single karst catchment and was based on a spatial survey rather than a temporal experiment. The relative importance of soil thickness, landform, and land use may therefore vary among karst regions with different lithology, climate, soil-development histories, and management practices. Future multi-catchment comparisons and repeated or long-term observations would help evaluate the broader applicability of the relationships identified in the Houzhaihe catchment. Overall, this study reveals contrasting spatial responses of STN concentration and STN stocks to topographic, geomorphic, and land-use gradients within a karst catchment and highlights the importance of soil thickness in determining how nitrogen concentration translates into area-based nitrogen stocks. Incorporating both STN concentration and STN stocks into a unified assessment framework is essential for improving the reliability of soil nitrogen-pool estimates and for optimizing farmland nitrogen management and ecological restoration strategies in karst regions.

## 5 Conclusions

This study revealed a pronounced spatial decoupling between STN concentration and STN stocks in the Houzhaihe karst catchment, highlighting the limitations of assessments based solely on nitrogen concentration. Farmland soils contained relatively high STN stocks. Although peak-cluster areas, forestland, and grassland had relatively high STN concentrations, the principal areas of soil nitrogen storage were located in farmland soils within karst depressions. This finding highlights the potential bias of assessments based solely on nitrogen concentration, which may overestimate the contribution of natural vegetation systems to regional soil nitrogen pools while underestimating the actual nitrogen stocks of farmland soils.

The structural equation model further identified soil thickness as a key structural factor underlying the decoupling between STN concentration and STN stocks. Soil thickness was the only factor that showed relatively strong but opposing associations with the two nitrogen metrics. By affecting effective soil mass and contributing to the vertical dilution of profile-scale nitrogen concentration, soil thickness provides an important structural basis for explaining the spatial differentiation of soil nitrogen pools. Given the characteristically shallow soils, extensive bedrock exposure, and pronounced spatial heterogeneity of karst landscapes, STN concentration alone cannot adequately represent the actual spatial pattern of soil nitrogen pools. Future soil nutrient assessments in karst regions should therefore integrate both nitrogen concentration and nitrogen stocks. These findings provide a scientific basis for improved soil nitrogen assessment, site-specific nitrogen management, and ecological restoration in karst landscapes.

## Data Availability

The raw data supporting the conclusions of this article will be made available by the authors, without undue reservation.
